# An emerging theoretical lens post global epidemic and turmoil: Psychological immunity^☆^

**DOI:** 10.1016/j.dialog.2025.100271

**Published:** 2025-12-23

**Authors:** Yi Xiao, Wei Wang, Xiping Hu

**Affiliations:** a Artificial Intelligence Research Institute, Shenzhen MSU-BIT University, 504 Main Building, Shenzhen 518172, China; b Artificial Intelligence Research Institute, Shenzhen MSU-BIT University, 512 Main Building, Shenzhen 518172, China; c Artificial Intelligence Research Institute, Shenzhen MSU-BIT University, 505 Main Building, Shenzhen 518172, China

**Keywords:** Psychological immunity, Biological immunity, Homeostasis, Ego

## Abstract

The concept of psychological immunity is not a new idea. In response to the escalating conflicts and chaos that characterizes this era, we conducted this study and reviewed psychological immunity theory from the perspective of psychiatry, psychology, and philosophy. We found that growing evidence has demonstrated the connection between biological and psychological immunity process. This indicates an integrated immunity system that has developed over time for survival in both physical and metaphysical sense. The process-oriented conceptualization of psychological immunity is presented, as well as its intra- and inter-mechanism. We also discussed research, practice, and policy potentials of this field and advocated for its protective application at individual and societal level. Limitations and future research were addressed accordingly.

## 1 Introduction

There is a growing consensus that this is an era of conflicts and unpredictability. Not long out of the pandemic, as described by the September 24th 2024 New York Times story *World leaders confront global turmoil at U.N.*, we entered “the shadow of war and turmoil in Europe, Africa and the Middle East”. As if an echo to this prediction, a new leadership took over Syria in December 2024. In April 2025, the United States raised tariffs to an unheard-of level. Post-pandemic turmoil has impacted lives of many. According to the United Nation International Labour Organization [1], the unemployment rate of 2024 was expected to increase, indicating the influence of Covid-19 and regional turmoil. In the United States, January 2025 unemployment statistics (4 %, 6.8 million people) were higher than that of the last year (3.7 %, 6.1 million; [2]). This growing unemployment anxiety has been further confounded by advances in technology. The development in Artificial Intelligence (AI), such as the DeepSeek, has expedited disruptive changes to current work environment. AI-enhanced transportation services, medical services, and educational services have already shifted the role of human-beings in a work setting. Some optimists like Elon Musk foresee a promising future with a universally high income after this replacement, whereas many worry about the choices left for humans. As Heraclitus put it, the only constant in our life is change. In this chaotic era, this uncertainty seems only to be amplified, and makes the ability to maintain stability, or psychological homeostasis, more and more important. In other words, having a sound psychological immunity against ever-increasing demands and changes, becomes a critical, practical need.

Psychological immunity is also an underlying theme in recent global policies. In Spring 2023, the Chinese government launched a national task force on student mental health, unprecedentedly endorsed by 17 departments, with the mission statement highlighting psychological immunity:*“love life, cherish life, have self-esteem and self-confidence, rational and stable-minded, optimistic and perseverant, unshakable regardless of honor or disgrace, and indomitable”.* The U.S. government launched the Unity Agenda, with tackling the mental health crisis as a critical goal [3]. Newly allocated funding on behavioral health and suicide prevention/mental health care exceeded $136 million dollars in September 2024 [4,5]. The National Health Services (NHS) England initiated an employment service with application advising and confidence building for people with mental illnesses, assisting over 40,000 people back to work [6]. In May 2025, the World Health Organization (WHO) Secretary-General highlighted promotion of mental health in the WHO 78th Assembly. These policy-led endeavors indicated that psychological immunity has gradually become salient in our lives.

Immunity is a system that protects organisms from pathogenic invasion [7]. It is composed of two major defense mechanisms: innate immunity and adaptive immunity. The immune response typically includes four steps: detection, communication, attack, and suppression. This biological process is being increasingly applied in a psychological context. To bridge the transfer and highlight this protection mechanism in current chaos, we conducted this study. Our study is three-fold. First, we reviewed psychological immunity theory from the perspective of psychiatry, psychology, and philosophy. Second, we reviewed the construct and mechanism of psychological immunity, and attempted to answer important conceptual questions. Third, in our discussion section, we proposed the potentials for research, practice, and policy, as well as limitations and future research.

## 2 Psychological immunity theory

There are three perspectives in the study of psychological immunity: psychiatry, psychology, and philosophy.

### 2.1 Psychiatric standpoint

The “inflammation hypothesis of mental illnesses” [8] is not a new thing. According to DeLisi, Weber, and Pert's literature review [9], Khoroshko was one of the earliest researchers to report the immunological underpinning of psychiatric disorders [10]. Arolt, Rothermundt, Peters, and Leonard reported that this field of study could be traced back to Hans Selye's benchmark study on stress in the 1930s [11]. These pioneers later expanded their exploration to neurotransmitter functions in stress responses, which led to the study on psychoneuroimmunology. Schizophrenia was among the first psychiatric disorders to be studied through the lens of immunology [12].

The focus of this field of study is to explore the immunological foundation of psychiatric disorders, typically in the context of severe ones such as schizophrenia and major depressive disorders. Major topics include identifying relevant biomarkers [[13], [14], [15], [16], [17]], examining the immunological response mechanism [[18], [19], [20], [21], [22], [23], [24]], and investigating relevant inter-generational hereditary phenomenon [[25], [26], [27], [28]]. Important biomarkers were identified. These biomarkers, found in blood and cerebrospinal fluid, include pro-inflammatory cytokines and proteins, neutrophil-lymphocyte ratios, NOD-like receptors containing pyrin and their inflammasomes [13,15,17,21,29]. Perry et al. conducted a mendelian randomization study using a series of immunological proteins/traits, and found that IL-6, IL-9, MCP-1, sIL-2Rα, and BDNF were associated with schizophrenia, with IL-6 being the most consistent and potentially associated with depression [21]. Their findings also negated the reverse causality hypothesis that psychiatric disorders would influence immunological proteins/traits. Yuan et al. reviewed 43 meta-analysis involving over 700 publications across eight major psychiatric disorders, and found that the following inflammation-related factors showed consistent patterns in multiple studies: CCL2, CRP, IL-4, IL-6, NGF, sIL-2R, sIL-6R, sTNF-R1, TNF-alpha, TFG-beta, and VEGF, denoting their diagnostic and treatment value [17]. Evidence from this body of research established the unique role of immunological responses in psychiatric disorders, indicating inflammation as the underlying pathogenic factor [29,30]. Chan et al. summarized this bi-directional process between the brain and the body: chronic stress suppressed the immunological function and led to inflammation in our body; this inflammation then induced the brain to initiate psychiatric symptoms [29]. Such mechanism was reported in a series of psychiatric and neuro-behavioral disorders, such as Depression [15,19,21], Schizophrenia [14,18,21], Tourette Syndrome [31], Attention Deficit/Hyperactivity Disorder [16,32], Bipolar Disorder [21,33,34], Autism [35], and Catatonia [36]. Similar changes were also reported in maternal and offspring samples [25,27].

This growing body of research is expected to influence the diagnosis and treatment of psychiatric disorders [37]. For example, Endres et al. proposed a subtype of Obsessive-Compulsive Disorder (OCD) named “autoimmune OCD” [38]. Fernandes et al. developed a machine learning algorithm using immunological and cognitive biomarkers to diagnose bipolar and schizophrenia [39]. The SSRI and SNRI can reduce the level of cytokines and regulate inflammatory pathways to alleviate depression [19]. Anti-inflammatory effect has been reported in existing psychiatric medications [19,40].

### 2.2 Psychological standpoint

Psychological homeostasis or emotional equilibrium is the underlying consensus in this field, and a sound psychological immunity can protect or restore this state and achieve normal psychological functioning [41,42]. In analogous to immunity or immune system, researchers developed the concept of psychological immunity or psychological/behavioral immune system [43,44]. These researchers believed there is a complementary psychological mechanism that generate behavioral responses to defend threats and reduce risk.

The first group of researchers focused on the connection between psychological status and immunological function. Stress is a highlighted threat to the “positive mood offset” [45], and impairs normal immunological functioning. In Segerstrom and Miller's benchmark study, stress response in the immune system is further categorized by the type of stressors: acute stressors, brief naturalistic stressors, and chronic stressors [46]. While acute and brief naturalistic stressors may induce adaptive changes, chronic stressors suppress the immune system. More recent researchers answered their call for further investigation into the psychological phenomena in stress, immunity, and disease responses. For example, Ilchmann-Diounou and Menard revealed that the presence of stress-induced intestinal dysfunction contributes to autoimmune diseases [47]. Westfall et al. found that the gut-brain-axis can prime immunity response against the chronic and recurrent stress, and alleviate associated depression and anxiety symptoms [48]. Bower and Kuhlman summarized immune-to-brain communication: the activation of immune system can impact positive and negative valence systems, social processes, cognition, and arousal at molecular, neural, and behavioral level, which in turn, leads to patients' self-report changes in psychological disorders symptoms [49]. This body of research supported the bidirectional connection between the psychological status and the immunological function.

The second group of researchers examined the conceptualization of psychological immunity and its theoretical structure. The psychological immunity is considered both a trait and a process, with the aim to maintain psychological homeostasis [43,50]. Its specific construct is differed depending on the combination of trait and process factors. Rachman posited that cognition is the core of psychological immunity, highlighting the underpinning cognitive appraisal process [51]. Besides the cognitive core, researchers also presented other dimensions, such as motivational, behavioral, and personality ones [43]. The psychological immunity is then conceptualized as an integrated system with cognitive, behavioral, and trait-like factors [52]. Along this line of conceptualization, the representative study is Oláh's psychological immunity model of three interactive subsystems with 16 factors [43]. The model is summarized in English as follows (pp. 140–141; [52]).

***Approach-belief subsystem:** optimism, coherence feeling, development feeling, control ability, personal source monitoring ability, social source monitoring ability.*

***Monitoring-creating-executing subsystem:** personal source mobilizing ability, personal source creating ability, social source mobilizing ability, social source creating ability, self-respect.*

***Self-regulating subsystem:** emotion control, excitability control, impulsivity control, persistence, synchrony ability.*

Building on Oláh's study [43], Attaran et al. underlined the context of interpersonal relationships and individuals' agency when facing challenges [50]. Three immunity responses were identified: threat recognition, response generation, and self-regulation. In their review of psychological constructs related to the immune system, Biela et al. proposed a four-factor psychological immunity structure: strength and will of meaningful life, sense of competence in coping, social support and proactivity, and autonomous goals [53,54]. Choochom proposed a five-factor model of psychological immunity: resilience, mindfulness, coping, hope, and self-reliance [55]. Aggab and Aouin established a six-factor model of psychological immunity: religious commitment, problem-solving ability, goal orientation, self-confidence, positive thinking, and emotional stability and self-regulation [56]. As a natural extension of this body of research, assessment instruments were developed. Representative instruments include the Psychological Immune Competence Inventory [43], the Self-Immunity Scale [55], and the Scale of Psycho-immunological Structure [53]. Other instruments were also used to assess psychological immunity. For example, Zábó et al. assessed psychological immunity using the Mental Health Test [57].

The third group of researchers applied this concept in practice and conducted intervention studies. Psychologists have been intrigued by the idea that chemical interventions, to certain extent, could be replaced by psycho-social ones. Kiecolt-Glaser and Glaser's groundbreaking study demonstrated that psychological interventions (e.g., relaxation, hypnosis, exercise, classical conditioning, self-disclosure, exposure to a phobic stressor, and cognitive-behavioral interventions) can enhance immunological functions [58]. Recent researchers reported similar results. Shields, Spahr, and Slavich conducted a systematic review and meta-analysis study of 56 unique randomized clinical trials, and found that psycho-social interventions, especially cognitive behavioral therapy, were associated with sustained improvements in immunological biomarkers, such as cytokines [59]. Bower et al. reported an anti-inflammatory effect at the gene expression level for mindfulness-based interventions [60]. Even nuances in the process have been investigated. Parraga, Parks, and Feldman underlined treatment adherence as a potential mediator in these studies [61].

### 2.3 Philosophical standpoint

Focused on self, identity, and individuality, the philosophical study on immunology has an established tradition [62]. As the renowned immunologist Burnet (p.17; [63]) put it, “Immunology has always seemed to me more a problem in philosophy than in practical science”. Swiatczak and Tauber outlined two conceptual shifts in immunology theory: from classical self/nonself framework to multifaceted reactivity model, from a defense mechanism view to an interactive mediation process [62]. The conceptualization of self and identity then diverged into very different paths, depending on the “individualist” or “collectivist” orientation that one has selected. This diversion, in turn, led to postmodern deconstruction of self-hood, and brought multi-faceted implications to the organization of our society [64].

Some researchers such as Ajana extended this social-political conversation. Ajana cautioned the “defending” and “sacrificing” logic rooted in the biological interpretation of immunity, and reaffirmed the importance of an accepting and empowering community [65]. Beyond the reductionism defense and strength perspective, Zach and Greslehner highlighted contextuality, regulation, and trade-offs in the interpretation of immunity [66]. Representative works in this field include: Tauber's *The Immune self: Theory or metaphor?* [67] and *Immunity: The evolution of an idea* [68], Esposito's *Immunitas: The protection and negation of life* [69], and Pradeu's *Philosophy of immunology* [70].

## 3 The construct and mechanism of psychological immunity

### 3.1 Construct

Maintaining psychological homeostasis is the agreed upon goal/function of psychological immunity in the research community [43,51,71,72]. However, researchers' views differed on how this goal is achieved. Some researchers believed the psychological immunity is a congregation of traits that could counter threats. For example, Albert-Lőrincz et al. (p. 104) argued that psychological immunity is “the total of personal traits” that protects an individual from harm and safeguards effective functioning [71]. More recent researchers referenced the biological immunity and conceptualized psychological immunity as a process [72,73]. For example, Sedikides [72] believed that psychological immunity is part of “an adaptation process” (p. 198) and “emotional equilibrium, or psychological well-being, is essential to biological adaptation” (p. 199). He contended that the psychological immunity process was originated from the evolution of the cognitive system, which was a result of human physical adaptation. This system, built on a highly sophisticated neuron-network, is able to perform high-level cognitive functions to maintain an individual's independence despite disrupting inputs [72]. Some researchers took a middle-ground and included both trait-like and process-like components in their conceptualization [43,50]. As aforementioned, Oláh proposed a three-level psychological immunity system that included the organization of different personality traits and behavioral processes [43].

We contend that the construct of psychological immunity is better explained in a context, in other words, through the achievement of its goal or function. Sedikides put forward the homeostatic model of identity protection to explain the function of psychological immunity [72]. He believed people's self-concept is constantly threatened by inputs that could deviate its positive functioning. For example, overly-negative comments can incur self-doubts and paralyze subsequent behaviors; overnight success may bring complacency and conceal potential risk. In line with the findings in positive psychology, emotions are able to influence people's behaviors [74]. The psychological immunity is then conceptualized as a dynamic mechanism evolved to protect the individual's self-concept and maintain a healthy, positive feeling about themselves [72]. Evidence from psychiatry and psychology studies supported the process-model of psychological immunity, highlighting the coordination of different components and processes. For example, Albert-Lőrincz et al. discovered that positive thinking, optimism, and feeling of control were associated with the flow experience in individual activities, whereas emotional control and self-regulation were associated with the flow experience during interactive activities [71].

#### 3.1.1 Differences between psychological immunity, resilience, and psychological strengths

Differed from resilience and psychological strengths, the concept of psychological immunity is retrieved from its biological counterpart [44]. The interactive cognition process that a person responds to psychological stimuli could share a biological foundation with the immune system [51]. Another notable distinction is that the psychological immunity includes a proactive learning mechanism [72], more sophisticated than the defend and rebound function associated with resilience. Studies on individuals exposed to traumatic events have revealed the intriguing phenomenon of “post-traumatic growth” [75]. Some trauma survivors have even become what Jung called “the wounded healer”. In these scenarios, resilience has transcended into an intricate transformation mechanism. It is no longer merely a passive receiver of psychological threats, but an active process embedded in an advanced cognitive system, as indicated by current researchers [72]. This process is not well-explained by the concept of resilience.

Similar to its biological counterpart, the psychological immunity is a structured system and a dynamic mechanism, rather than a simple compilation of different psychological strengths or traits. One or several psychological strengths could be employed to resolve a challenge, representing a liner problem-solving logic. Nevertheless, from the viewpoint of psychological immunity, a systemic perspective needs to be adopted in the face of challenges. The focus is the nature and degree of interactions among different bio-psychological processes. The actual impact is rarely one-directional, such as removal of unwanted negative feelings. Instead, it can entail simultaneous activation and deactivation. In experience, we may encounter “mixed feelings”, which is better explained within the psychological immunity framework.

### 3.2 Mechanism

We posit that the psychological immunity system functions via two lines of mechanisms: intra-mechanism and inter-mechanism.

#### 3.2.1 Intra-mechanism: How does psychological immunity operate?

The psychological immunity operates with subsystems analogous to innate immunity and adaptive immunity in the biological domain [42]. The two subsystems differ in three aspects: origin (gain through evolution or acquired through experience), specificity (undifferentiated to all kinds of threat or threat-specific), and temporality (pre-threat or post-threat) [42]. Just as the biological immunity produces leukocytes into our body, the psychological immunity generates narratives into our cognition to counter psychological threats [72]. These narratives were grouped into preemptive and reparative types, with the former executing the function of innate immunity and the later for adaptive immunity [72]. Preemptive narratives are general and could be retrieved across various situations, whereas reparative narratives are formulated and summoned for specific threats to an individual's self-concept.

#### 3.2.2 Inter-mechanism: Connection between psychological immune system and biological immune system

With a growing amount of evidence substantiating the connection between these once considered separated systems, the psychological self and immunological self are now believed mutually generative but not isolated [73]. In other words, the discrimination of self/non-self and subsequent tolerate/defense responses is an integrated process, only to be derived into psychological or immunological pathways. Booth and Ashbridge proposed an extended framework of the immune system including psychological, neuroendocrine, and immunological subsystems [73]. With three subsystems operating in parallel and interrelatedly, the structure and function of the immune system are inherently multidimensional in the role of “somatopsychic determination of self” (pp. 15–17). In a similar vein, Sedikides [72] claimed that biological and psychological immunity are “components of a coordinated, adaptive, harm protection system in humans” (p. 198), and “neither biological nor psychological homeostasis can be achieved, if either aspect of the body's maintenance system malfunctions” (p. 199). As a result, the biological immune system is conceptualized as a system constituted of cellular components, whereas the psychological immune system is its counterpart made up of beliefs, narratives, and projections [72]. This inter-mechanism, aligned with the unity of opposites principle, underscores the complementary roles of psychological and biological immune system in maintaining overall well-being.

**Fig. 1:**
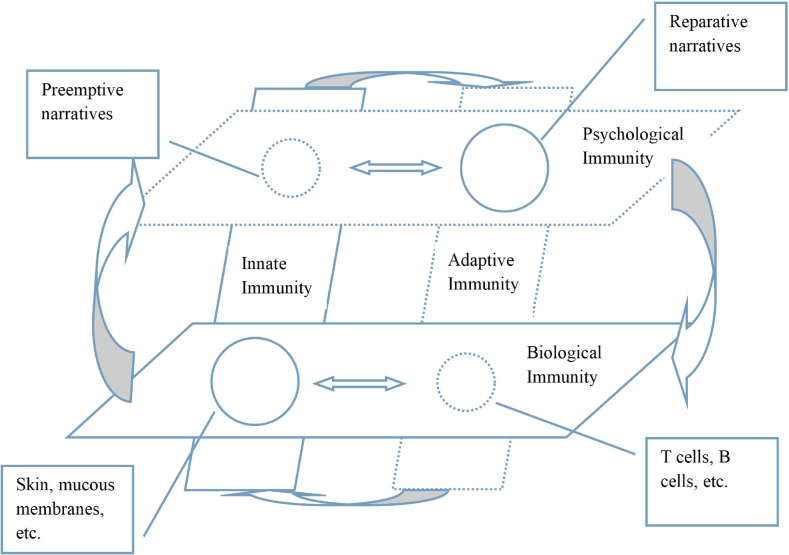
Conceptual model of the integrated immunity system.

Overall, our proposed conceptual model of the integrated immunity system (Fig. 1) draws on relevant theoretical and empirical endeavors across the fields of psychiatry, psychology, and philosophy. Each discipline contributes uniquely to the current model. Psychiatric studies established the biological foundation of the immunity system and provided a core framework to understand human immunity, as represented by two structural designs in the model: the relative positional hierarchy of biological and psychological immunity, and horizontal (inter-system) and vertical (intra-system) groupings. Psychological studies, however, uncovered the intricate connections during biological and psychological immunity processes, and highlighted an integrated immunity system in the face of threats, as visualized by the inter- and intra- system interactions in the model. Inspired by philosophical explorations, the unity of opposites principle was employed --- solid-lined and dented-lined shapes represent opposing but complementary elements within the system, with circle sizes denoting hypothetical quantitative variations. In sum, this model demonstrates a holistic human hazard prevention system, with the core function of self/non-self discrimination underpinning all immunity processes.

## 4 Discussion

### 4.1 The era requires advancement of understanding in human psychology

The remarkable progress in the physical realm demands commensurate advancements in the metaphysical sphere to maintain equilibrium. The pandemic, war, AI, ambitious expedition to the outer space, among other momentous developments, have reminded us that changes are taking place at a breathtaking and unprecedented pace. Maintaining a sense of consistency and stability, naturally, attracts growing attention. This line of inquiry eventually leads us to the essence of psychological immunity. *Why do we have this mechanism? What is this system protecting us from?* We propose that just as we have to set biological boundaries with the environment to safeguard physical health, we also have to set psychological ones to retain our self-hood, without which our existence seems to be lost in the metaphysical limbo. The underlying anxiety of our era is probably existential in nature: no one is irreplaceable across any kind of setting or relationship, nothing cannot be changed unexpectedly in a sudden. In addition, the advances in technology unveil the potential of an intelligence beyond human cognition, compromising our superior status among living creatures. The existential anxiety of being overwhelmed by these changes is imminent. The human psychology is possibly the final defense of human-hood, just as Issac Asimov subtly hinted in the *Second Foundation*.

An important discussion should be directed to the nature of psychiatric disorders and suicide. We are drawn to question if mental illnesses such as depression and anxiety are results of an impaired psychological immunity system weakened by “psychological pathogens”. Intuitively, suicide becomes the ultimate psychological immunity failure [51], or an extreme immunological rejection response that aims to annihilate the “unwanted self”. As Bell [76] referenced Freud's (p. 252; [77]) comment: “The ego can kill itself only if it can treat itself as an object.” The psychological immune system also provides a framework to understand how the ego system, propelled by psychological drives, defines what is self or non-self through a negotiation between the *superego* and the *id*. Indeed, researchers cautioned the reductionist stance when making analogies between psychological immunity and biological immunity [42]. We earnestly present this parallelism and advocate for revisiting Freud's work on psychological drives and ego system for inspiration.

### 4.2 Potentials for research, practice, and policy

#### 4.2.1 Research

The biological mechanism of psychological immunity awaits further exploration. Arolt et al. expressed skepticism regarding the direct translation of animal study results to human-beings in this field, pointing out the apparent difference between animal and human immune systems [11]. There is also an absence of subjective human experience in the animal immunological framework [73]. This is perhaps a particularly significant absence, given the evidence of an integrated immunity process [49,73]. Careful and creative selection of human participants is necessary. The biological validity of statistically significant results in this field also raised concerns [11]. Advances in research methods and designs that can yield more clinically significant results were advocated [11]. These questions, proposed over twenty years ago, still call for compelling answers. Future researchers need to monitor biological and psychological responses simultaneously to understand the entire immunity process. This integrated approach will present a more comprehensive picture of the entire immunity process, potentiating novel discoveries in their connection and mechanism.

Further exploration of psychological immunity treatment mechanism, design and delivery, and effectiveness in different populations are also needed. Researchers can explore the anti-inflammatory effect of psychotherapy. This may include examining active ingredients in talk therapy, such as how therapeutic alliances can improve inflammatory levels. Additionally, they can explore how psychological interventions operate at the biological level. This may involve comparing this process to chemical treatment and examining the dose-effect relationship. Longitudinal studies that assess long-term effects are also needed. There are also potentials for subfields. For example, “affective immunology” [78] and “language leaner immunity” [50] demonstrated exciting opportunities in exploring the psychological immunity construct and its extension. Inspired by Wittmann et al.'s work on social decision [79], we tentatively suggest that mathematics may be necessary to approximate the essence of psychological immunity: its mathematical expression may be an interactive and generative algorithm system, building on core psychological constructs within the conceptual spaces framework [80].

#### 4.2.2 Practice

Precision treatments designed to alleviate inflammatory responses in clinical population were repeatedly suggested, signaling pioneering opportunities for treatment advancement [34,37,81]. In practice, the application could be quite complicated. Warner-Schmidt, Vanover, Chen, Marshall, and Greengard reported an antagonizing effect of anti-inflammatory medicine and SSRIs, cautioning the drug combination of these two types of medications [82]. Some researchers suggested using anti-inflammatory treatments for patients with psychiatric disorders and severe inflammation, especially those not responding to traditional psychiatric medications [81].

More over, psychological immunity interventions can be used proactively as “psychological vaccination”. For example, in 2013, to reduce suicide rate and the risk of Post Traumatic Stress Disorder among marines, the United States Marine Corps piloted the Mindfulness-Based Mind Fitness Training before deployment. Mindfulness and other forms of meditation services have become widely available in the military. To promote people's psychological immunity to misinformation, Van der Linden proposed the use of prebunking techniques, such as informing people of possible manipulation, as well as giving examples of misinformation and teach people how to refute [83]. Dai and Smith [84] reviewed resilience to depression literature and recommended the use of natural stress-vaccination (e.g., controlled stress scenario provided by parents) or clinical vaccination (e.g., cognitive behavioral therapy). This individual effect can be aggregated: an individual's risk is significantly reduced if people around were able to achieve psychological immunity. Basol et al. coined the term “psychological herd immunity” to describe this phenomenon [85]. In this specific scenario, people became more alert to Covid-19 misinformation after prebunking interventions, and subsequently refrained from spreading the misinformation. Immunization has long been regarded as “a global health success story”, preventing millions of deaths every year [86]. These psychological updates may potentially refine global health.

#### 4.2.3 Policy

We advocate for applying psychological immunity in the design and implementation of policies. Policies that foster “psychological immunity empowering culture” may help us build a more inclusive environment that ultimately benefits the vast majority, such as promoting prolonged life expectancy [87]. In the same way, the global mental health crisis can potentially be alleviated if we together build a stronger defense. Furthermore, the lens of psychological immunity offers a systemic perspective, giving insights into how certain policies interact, or even cancel out their effects. For example, while welfare can relieve people's financial stress, over compensation is often considered to reduce return-to-work motivation and increase the risk of dependency.

We contend that our policies should strongly encourage human agency. In our defining self-hood, we face the blatant moral question of whether life is only worthwhile when it is happy. Excessively pursuing happiness could potentially hinder our development of psychological immunity and forfeit the gravity of this valuable process. Bravery, determination, devotion, and perseverance are all indispensable qualities to an individual's self-actualization, which are learned the best in difficulty times. Evolution has endowed us the built-in immunity mechanism to detect and confront malice in the environment. Our biological and psychological immunity systems are normally on auto-pilot mode, but open to and require manual reinforcement in response to different forms of malice. We are obligated to utilize and strengthen these mechanisms.

On a further note, our study aligns with the WHO's endeavors in this era of turbulence. The WHO upholds the Sustainable Development Goals (SDGs) relevant to global health. Our study contributes to the following SDGs: SDG 3 (Ensure healthy lives and promote well-being for all at all ages), SDG 10 (Reduce inequality within and among countries), SDG 11 (Make cities and human settlements inclusive, safe, resilient and sustainable), and SDG 16 (Promote peaceful and inclusive societies for sustainable development, provide access to justice for all and build effective, accountable and inclusive institutions at all levels). In addition, the WHO's Fourteenth General Programme of Work 2025–2028 (GPW 14) outlines six objectives that “advance health equity and build resilience in a turbulent world” (p. vii; [88]). Our study addresses three of the GPW 14 Objectives: Objective 2 (Address determinants of health and root causes of ill health), Objective 5 (Prevent, mitigate, and prepare for health risks from all hazards), and Objective 6 (Rapidly detect and sustain response to health emergencies). To illustrate, the concept of psychological immunity enhances the current understanding of the natural human hazard prevention system (i.e., the immune system) against sustained and sudden threats. It also reframes the conceptualization of health and illness as an interactive process between threats and an integrated immunity system. This concept, inherently carries cross-disciplinary value at both individual and societal levels, can be transferred and generalized across these disciplines and diverse populations. For example, psychological immunity interventions may be adopted to supplement, enhance, and even --- to some extent --- replace traditional medical approaches, opening opportunities for more affordable, less restrictive, and non-intrusive treatments. We also wish to emphasize its special value to underserved populations worldwide, as they lack resources to cope with sudden changes and are particularly vulnerable amid instability. The promotion of psychological immunity contributes to the well-being of everyone, embodying the spirit of beneficence, equality, and inclusion.

### 4.3 Limitations and future research

To address the timely progress of psychological immunity research in the context of post-pandemic turmoil, similar to Graham et al.'s methods [89], except for several representative pre-pandemic works, we included studies published on and after the onset of the pandemic (i.e., year 2019), with a focus on those from leading academic journals or publishers. Google Scholar was used to identify relevant literature in the field of psychiatry, psychology, and philosophy. Search keywords included psychological immunity, psychoimmunology, psychoneuroimmunology, psychiatry and immunology, psychology and immunology, and philosophy and immunology. Additionally, three articles from neurology, computer science, and public health were incorporated to facilitate discussions on psychological immunity. This process yielded a total of 80 studies (Table 1), including 36, 32, and 9 in the subfields of psychiatry, psychology,and philosophy, respectively. Notably, our study did not employ a systematic review framework and our literature list is not exhaustive. Future researchers can enhance methodological rigor by employing such frameworks, adhering to the PRISMA guidelines. Another limitation is that our study focused on immediate outcomes rather than using a prospective design to evaluate retention and application of knowledge related to psychological immunity over time. Future researchers can extend this study by analyzing samples from clinical populations (e.g., trauma survivors) or conducting qualitative case studies to explore the phenomenology of psychological immunity among people with chronic conditions.

**Table 1:** Summary of literature used in review and discussion.

Number	Authors	Type of Work	Title	Field of Study	Year	Journal/ Publisher	Reference Number
1	Martone	Research article	The inflammation hypothesis and mental illness	Psychiatry	2019	J of Clin Psychiatry and Neurosci	8
2	DeLisi, Weber, and Pert	Research article	Are there antibodies against brain in sera from schizophrenic patients? Review and prospectus	Psychiatry	1985	Biol Psychiatry	9
3	Khoroshko	Dissertation	Reactii Zivotnogo Organizma na Vvedenie Nervnoi Tkani [Reactions of the animal organism to the introduction of nervous tissue]	Psychiatry	1912	Moscow Publisher	10
4	Arolt, Rothermundt, Peters, and Leonard	Research article	Immunological research in clinical psychiatry	Psychiatry	2002	Mol Psychiatry	11
5	Lehmann-Facius	Research article	Über die Liquordiagnose der Schizophrenien [About CSF Diagnosis of Schizophrenia]	Psychiatry	1937	Klin Wochenschr	12
6	Bhikram and Sandor	Research article	Neutrophil-lymphocyte ratios as inflammatory biomarkers in psychiatric patients	Psychiatry	2022	Brain, Behav, and Immun	13
7	Ermakov, Melamud, Buneva, and Ivanova	Research article	Immune system abnormalities in schizophrenia: an integrative view and translational perspectives	Psychiatry	2022	Front in Psychiatry	14
8	Pandey, Zhang, Sharma, and Ren	Research article	Innate immunity receptors in depression and suicide	Psychiatry	2021	J of Psychiatry and Neurosci	15
9	Schnorr, Siegl, Luckhardt, and others	Research article	Inflammatory biotype of ADHD is linked to chronic stress: a data-driven analysis of the inflammatory proteome	Psychiatry	2024	Transl Psychiatry	16
10	Yuan, Chen, Xia, Dai, and Liu	Research article	Inflammation-related biomarkers in major psychiatric disorders	Psychiatry	2019	Transl Psychiatry	17
11	Benros and Mortensen	Book chapter	Role of infection, autoimmunity, atopic disorders, and the immune system in schizophrenia: evidence from epidemiological and genetic studies	Psychiatry	2019	Springer	18
12	Dionisie, Filip, Manea, Manea, and Riga	Research article	The anti-inflammatory role of SSRI and SNRI in the treatment of depression: a review of human and rodent research studies	Psychiatry	2021	Inflammopharmacology	19
13	Gangadin, Enthoven, van Beveren, Laman, and Sommer	Research article	Immune dysfunction in schizophrenia spectrum disorders	Psychiatry	2024	Annu Rev. of Clin Psychol	20
14	Perry, Upthegrove, Kappelmann, Jones, Burgess, and Khandaker	Research article	Associations of immunological proteins/traits with schizophrenia, major depression and bipolar disorder: a bi-directional two-sample mendelian randomization study	Psychiatry	2021	Brain, Behav, and Immun	21
15	Ravi, Miller, and Michopoulos	Research article	The immunology of stress and the impact of inflammation on the brain and behaviour	Psychiatry	2021	BJPsych Adv	22
16	Shimo, Cathomas, Lin, and others	Research article	Social stress induces autoimmune responses against the brain	Psychiatry	2023	Proc of the Natl Acad of Sci	23
17	Wang, Zhu, Zhang, and others	Research article	Causal role of immune cells in schizophrenia	Psychiatry	2023	BMC Psychiatry	24
18	Conway and Brown	Research article	Maternal immune activation and related factors in the risk of offspring psychiatric disorders	Psychiatry	2019	Front in Psychiatry	25
19	Hantsoo, Kornfield, Anguera, and Epperson	Research article	Inflammation: a proposed intermediary between maternal stress and offspring neuropsychiatric risk	Psychiatry	2019	Biol Psychiatry	26
20	Mueller, Scarborough, Schalbetter, and others	Research article	Behavioral, neuroanatomical, and molecular correlates of resilience and susceptibility to maternal immune activation	Psychiatry	2021	Mol Psychiatry	27
21	Pouget, Schizophrenia Working Group of the Psychiatric Genomics Consortium, Han, and others	Research article	Cross-disorder analysis of schizophrenia and 19 immune-mediated diseases identifies shared genetic risk	Psychiatry	2019	Hum Mol Genetics	28
22	Chan, Poller, Swirski, and Russo	Research article	Central regulation of stress-evoked peripheral immune responses	Psychiatry	2023	Nat Rev. Neurosci	29
23	Scangos, State, Miller, Baker, and Williams,	Research article	New and emerging approaches to treat psychiatric disorders	Psychiatry	2023	Nat Med	30
24	Martino, Johnson, and Leckman	Research article	What does immunology have to do with normal brain development and the pathophysiology underlying Tourette syndrome and related neuropsychiatric disorders?	Psychiatry	2020	Front in Neurol	31
25	Misiak, Wojta-Kempa, Samochowiec, and others	Research article	Peripheral blood inflammatory markers in patients with attention deficit/hyperactivity disorder (ADHD)	Psychiatry	2022	Prog in Neuro-psychopharmacology and Biol Psychiatry	32
26	Pereira, Oliveira, Silva, Madeira, Pereira, and Cruz	Research article	Inflammation in Bipolar Disorder (BD): identification of new therapeutic targets	Psychiatry	2021	Pharmacological Res	33
27	Rantala, Luoto, Borráz-León, and Krams	Research article	Bipolar disorder: an evolutionary psychoneuroimmunological approach	Psychiatry	2021	Neurosci & Biobehav Rev	34
28	Matta, Hill-Yardin, and Crack	Research article	The influence of neuroinflammation in autism spectrum disorder	Psychiatry	2019	Brain, Behav, and Immun	35
29	Rogers, Pollak, Blackman, and David	Research article	Catatonia and the immune system: a review	Psychiatry	2019	The Lancet Psychiatry	36
30	Bennett and Molofsky	Research article	The immune system and psychiatric disease: a basic science perspective	Psychiatry	2019	Clin and Exp Immun	37
31	Endres, Pollak, Bechter, and others	Research article	Immunological causes of obsessive-compulsive disorder: Is it time for the concept of an “autoimmune OCD” subtype?	Psychiatry	2022	Transl Psychiatry	38
32	Fernandes, Karmakar, Tamouza, and others	Research article	Precision psychiatry with immunological and cognitive biomarkers	Psychiatry	2020	Transl Psychiatry	39
33	Qian, Zhong, Zhang, Qiu, and Tan	Research article	Fluoxetine mitigates depressive-like behaviors in mice via anti-inflammation and enhancing neuroplasticity	Psychiatry	2024	Brain Res	40
34	Booth and Ashbridge	Book chapter	Implications of psychoimmunology for models of the immune system	Psychiatry	2019	CRC Press	73
35	Drevets, Wittenberg, Bullmore, and Manji	Research article	Immune targets for therapeutic development in depression: towards precision medicine	Psychiatry	2022	Nat Revi Drug Discov	81
36	Warner-Schmidt, Vanover, Chen, Marshall, and Greengard	Research article	Antidepressant effects of selective serotonin reuptake inhibitors (SSRIs) are attenuated by antiinflammatory drugs in mice and humans	Psychiatry	2011	Proc of the Natl Acad of Sci	82
37	Kavčič, Avsec, and Zager Kocjan	Research article	Psychological functioning of Slovene adults during the COVID-19 pandemic: Does resilience matter?	Psychology	2020	The Psychiatr Q	41
38	Vaz, Mata, and Critcher	Research article	Analogies offer value through the struggle to make them work: making sense of the psychological immune system	Psychology	2021	Psychol Inq	42
39	Oláh	Book	Anxiety, coping, and flow: empirical studies in interactional perspective	Psychology	2005	Trefort Press	43
40	Schaller, Murray, and Hofer	Research article	The behavioral immune system and pandemic psychology	Psychology	2022	Eur Rev. of Soc Psychol	44
41	Diener, Kanazawa, Suh, and Oishi	Research article	Why people are in a generally good mood	Psychology	2015	Personal and Soc Psychol Rev	45
42	Segerstrom and Miller	Research article	Psychological stress and the human immune system: a meta-analytic study of 30 years of inquiry	Psychology	2004	Psychol Bull	46
43	Ilchmann-Diounou and Menard	Research article	Psychological stress, intestinal barrier dysfunctions, and autoimmune disorders: an overview	Psychology	2020	Front in Immun	47
44	Westfall, Caracci, Estill, Frolinger, Shen, and Pasinetti	Research article	Chronic stress-induced depression and anxiety priming modulated by gut-brain-axis immunity	Psychology	2021	Front in Immun	48
45	Bower and Kuhlman	Research article	Psychoneuroimmunology: an introduction to immune-to-brain communication and its implications for clinical psychology	Psychology	2023	Annu Rev. of Clin Psychol	49
46	Attaran, Ghonsooly, Hosseini Fatemi, and Shahriari	Research article	Immunology of language learners: a social psychological perspective	Psychology	2019	Interchange	50
47	Rachman	Research article	Invited essay: cognitive influences on the psychological immune system	Psychology	2016	J of Behav Thera and Exp Psychiatry	51
48	Kaur and Som	Research article	The predictive role of resilience in psychological immunity	Psychology	2020	Int J Curr Res and Rev	52
49	Biela, Špajdel, Śliwak, and others	Research article	Odporność psychiczna: jej struktura ikonstrukcja Skali Struktury Psychoimmunologicznej (SPS) [Mental resilience: Its structure and the construction of the Scale of Psychoimmunological Structure (SPS)]	Psychology	2013	Czasopismo Psychologiczne	53
50	Biela, Spajdel, Sliwak, Bartczuk, Wiechetek, and Zarzycka	Research article	The scale of psycho-immunological structure: assessing factorial invariance in Poland and Slovakia	Psychology	2015	Studia Psychologica	54
51	Choochom	Research article	Development of self-immunity scale	Psychology	2013	ATINER'S Conference Paper Series	55
52	Aggab and Aouine	Research article	Construction of a psychological immunity scale for middle school students	Psychology	2025	Soc Studies and Res J	56
53	Zábó, Erát, Gonda, and others	Research article	Psychological immunity: a new mental health test for psychiatric samples	Psychology	2024	Eur Psychiatry	57
54	Kiecolt-Glaser and Glaser	Research article	Psychoneuroimmunology: Can psychological interventions modulate immunity?	Psychology	1992	J of Consult and Clin Psychol	58
55	Shields, Spahr, and Slavich	Research article	Psychosocial interventions and immune system function: a systematic review and meta-analysis of randomized clinical trials	Psychology	2020	JAMA Psychiatry	59
56	Bower, Crosswell, Stanton, and others	Research article	Mindfulness meditation for younger breast cancer survivors: a randomized controlled trial	Psychology	2015	Cancer	60
57	Parraga, Parks, and Feldman	Book chapter	Treatment adherence: connecting psychology with immune treatment	Psychology	2025	Springer Nature Switzerland	61
58	Albert-Lőrincz, Albert-Lőrincz, Kádár, Krizbai, and Lukács-Márton	Research article	Relationship between the characteristics of the psychological immune system and the emotional tone of personality in adolescents	Psychology	2011	New Edu Rev	71
59	Sedikides	Research article	Self-construction, self-protection, and self-enhancement: a homeostatic model of identity protection	Psychology	2021	Psychol Inq	72
60	Fredrickson	Research article	The role of positive emotions in positive psychology: the broaden-and-build theory of positive emotions	Psychology	2001	American Psychol	74
61	Jayawickreme and Blackie	Research article	Post-traumatic growth as positive personality change	Psychology	2014	Eur J of Personal	75
62	Bell	Research article	Who is killing what or whom? Some notes on the internal phenomenology of suicide	Psychology	2001	Psychoanal Psychother	76
63	Freud	Book chapter	Mourning and Melancholia	Psychology	1917	Hogart Press	77
64	Graham-Engeland	Research article	Moving towards affective immunology: legacy and future directions	Psychology	2024	Comprehensive Psychoneuroendocrinology	78
65	Van der Linden	Book	Foolproof: Why misinformation infects our minds and how to build immunity	Psychology	2023	WW Norton & Company	83
66	Dai and Smith	Research article	Resilience to depression: implication for psychological vaccination	Psychology	2023	Front in Psychiatry	84
67	Basol, Roozenbeek, Berriche, Uenal, McClanahan, and Linden	Research article	Towards psychological herd immunity: cross-cultural evidence for two prebunking interventions against COVID-19 misinformation	Psychology	2021	Big Data & Soc	85
68	Oláh, Nagy, and Tóth	Research article	Life expectancy and psychological immune competence in different cultures	Psychology	2010	Empir Text and Cult Res	87
69	Swiatczak and Tauber	Book chapter	Philosophy of immunology	Philosophy	2020	The Stanford Encyclopedia of Philosophy	62
70	Burnet	Book chapter	A Darwinian approach to immunity	Philosophy	1965	Acad Press	63
71	Tauber	Research article	Immunity in context: science and society in dialogue	Philosophy	2016	Theoria: An Int J for Theo, Hist and Found of Sci	64
72	Ajana	Research article	Immunitarianism: defense and sacrifice in the politics of Covid-19	Philosophy	2021	Hist and Philos of the Life Sci	65
73	Zach and Greslehner	Research article	Understanding immunity: an alternative framework beyond defense and strength	Philosophy	2023	Biol & Philos	66
74	Tauber	Research article	The Immune self: Theory or metaphor?	Philosophy	1994	Immun Today	67
75	Tauber	Book	Immunity: the evolution of an idea	Philosophy	2017	Oxford University Press	68
76	Esposito	Book	Immunitas	Philosophy	2011	Polity Press	69
77	Pradeu	Book chapter	Philosophy of immunology	Philosophy	2020	Cambridge University Press	70
78	Wittmann, Lin, Pan, and others	Research article	Basis functions for complex social decisions in dorsomedial frontal cortex	Neurology	2025	Nat	79
79	Fierz	Research article	Conceptual spaces of the immune system	Computer Science	2016	Front in Immun	80
80	World Health Organization	Public information	Vaccines and immunization	Public Health	2024	https://www.who.int/health-topics/vaccines-and-immunization/#tab=tab_1	86

## 5 Conclusion

In retrospect, our original intention was protecting the individual and the general public in the midst of this violent, unremitting change. We quickly realized that the answer might already lie within us, similar to Hari Seldon found Trantor. Much to our surprise, the psychological immunity is not at all a rhetorical shortcut, and people are equipped with an immunity system including both biological and psychological mechanisms. The psychological immunity is a promising, exciting field to be furthered. The chaos that characterizes this era may even make it a desirable timing. To close with another analogy, we hope this study is part of the “social immunity process”, and advocate for creative employment of psychological immunity.

## Credit authorship contribution statement

**Yi Xiao:** Writing – original draft, Conceptualization. **Wei Wang:** Writing – review & editing. **Xiping Hu:** Writing – review & editing.

## Funding

YX has been supported by the Post-doc Grant of Shenzhen, China.

## Declaration of competing interest

The authors declare the following financial interests/personal relationships which may be considered as potential competing interests:

Yi Xiao reports financial support was provided by Shenzhen Municipal Human Resources and Social Security Bureau. If there are other authors, they declare that they have no known competing financial interests or personal relationships that could have appeared to influence the work reported in this paper.
